# Supplementary material for: Exploration of structure-activity relationships for the SARS-CoV-2 macrodomain from shape-based fragment linking and active learning
Source: Sci Adv. 2025 May 28;11(22):eads7187. doi: 10.1126/sciadv.ads7187 (PMC12118597; doi:10.1126/sciadv.ads7187)
Supplement: Supplementary file 1 — Figs. S1 to S7 Tables S1 and S3 Legend for table S2 [file sciadv.ads7187_sm.pdf]

Science Advances
AAAS

Supplementary Materials for

Exploration of structure-activity relationships for the SARS-CoV-2 macrodomain from shape-based fragment linking and active learning

Galen J. Correy *et al.*

Corresponding author: James S. Fraser, jfraser@fraserlab.com; W. Patrick Walters, pwalters@relaytx.com; Adam R. Renslo, adam.renslo@ucsf.edu

Sci. Adv. **11**, eads7187 (2025)
DOI: 10.1126/sciadv.ads7187

The PDF file includes:

Figs. S1 to S7
Tables S1 and S3
Legend for table S2

Other Supplementary Material for this manuscript includes the following:

Table S2

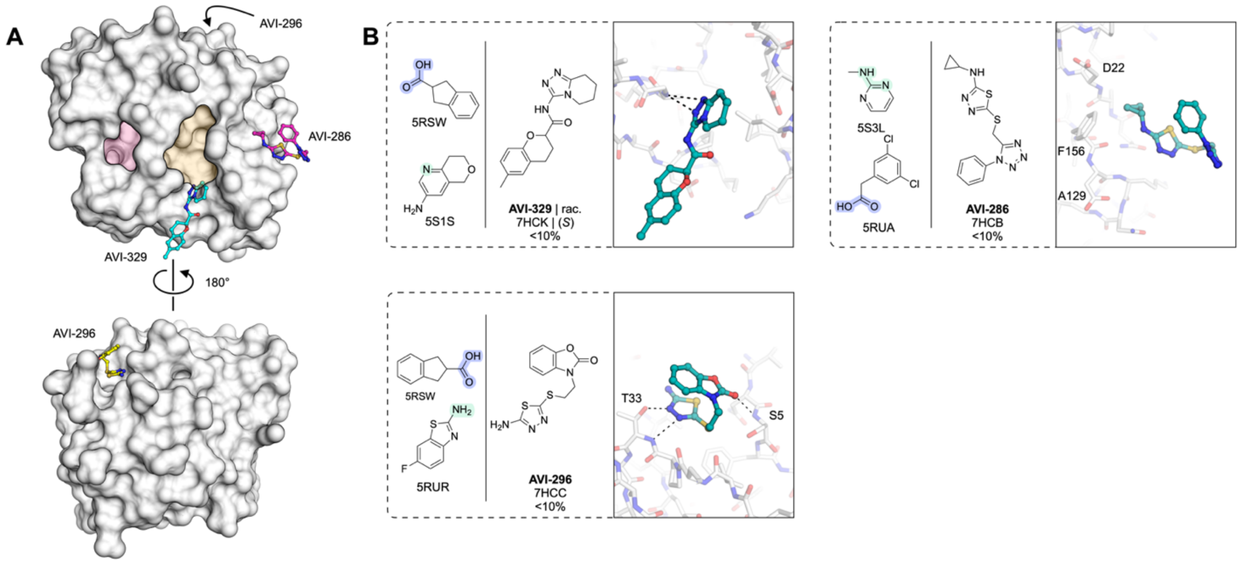

Fig. S1.

Three hits from the FrankenROCS pipeline were identified binding outside of the Mac1 adenosine site. (**A**) Surface view of Mac1 with the adenosine (yellow) and terminal ribose sites (red) shaded. (**B**) Chemical structures of the hits shown in (**A**). X-ray crystal structures are shown with hydrogen bonds shown as dashed black lines. Results for the preliminary HTRF screen (% competition at 200 µM, mean of three technical replicates) are indicated.

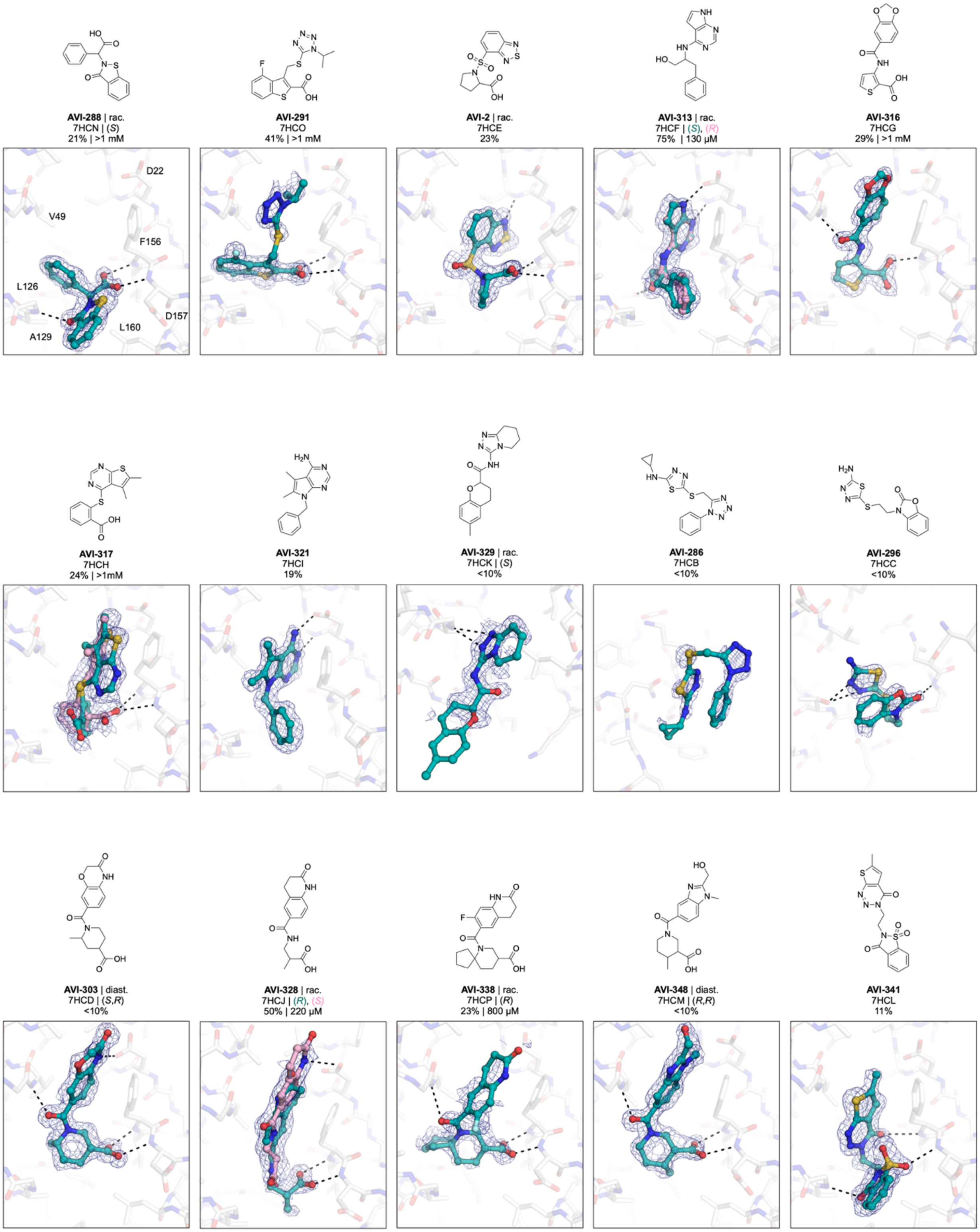

AVI-288 | rac.
7HCN | (S)
21% | >1 mM
D22
V49
F156
L126
D157
L160
A129
AVI-291
7HCO
41% | >1 mM
AVI-2 | rac.
7HCE
23%
AVI-313 | rac.
7HCF | (S), (R)
75% | 130 µM
AVI-316
7HCG
29% | >1 mM
AVI-317
7HCH
24% | >1mM
AVI-321
7HCI
19%
AVI-329 | rac.
7HCK | (S)
<10%
AVI-286
7HCB
<10%
AVI-296
7HCC
<10%
AVI-303 | diast.
7HCD | (S,R)
<10%
AVI-328 | rac.
7HCJ | (R), (S)
50% | 220 µM
AVI-338 | rac.
7HCP | (R)
23% | 800 µM
AVI-348 | diast.
7HCM | (R,R)
<10%
AVI-341
7HCL
11%

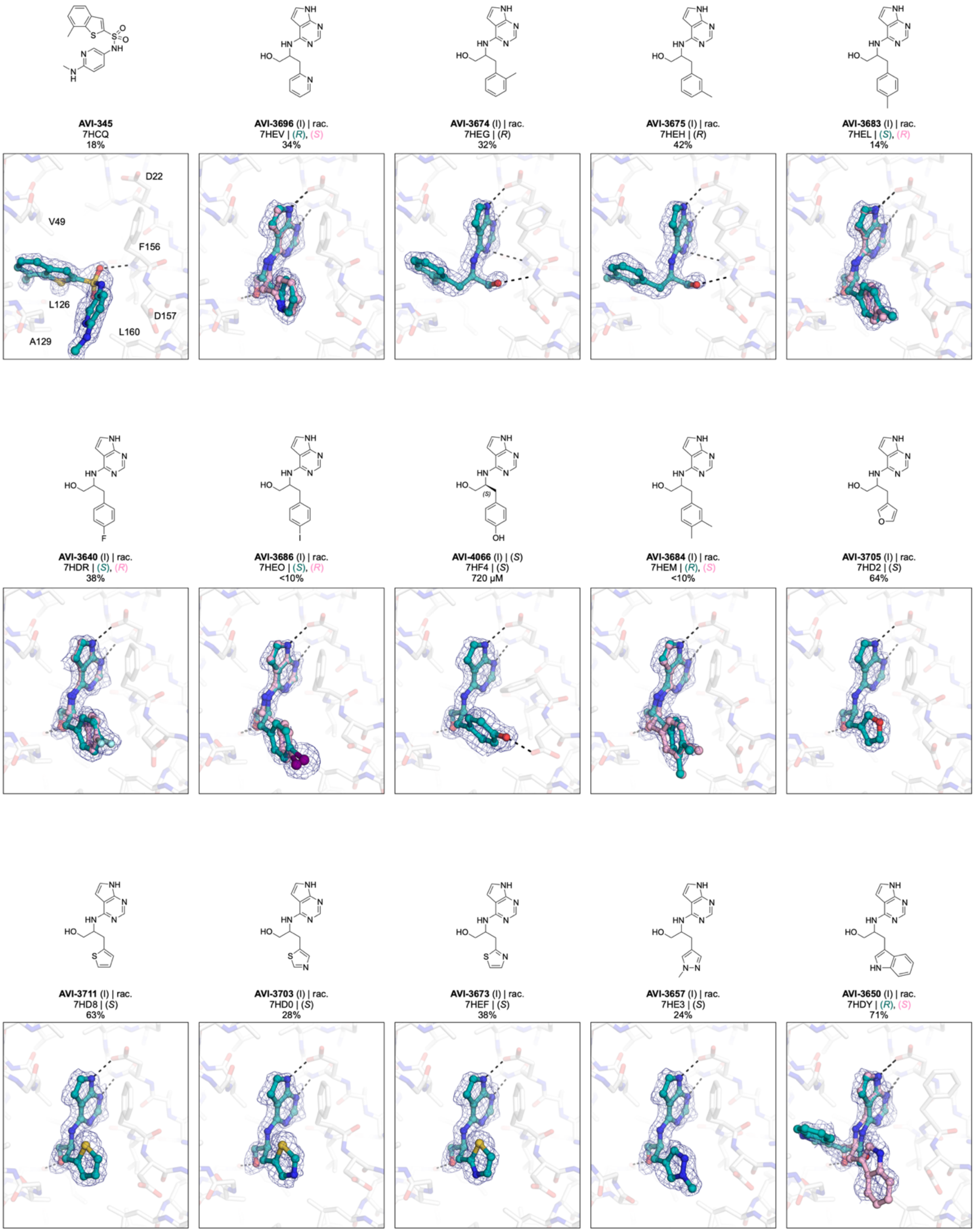
AVI-345
7HCQ
18%
D22
V49
F156
L126
D157
A129
L160
AVI-3696 (I) | rac.
7HEV | (R), (S)
34%
AVI-3674 (I) | rac.
7HEG | (R)
32%
AVI-3675 (I) | rac.
7HEH | (R)
42%
AVI-3683 (I) | rac.
7HEL | (S), (R)
14%
AVI-3640 (I) | rac.
7HDR | (S), (R)
38%
AVI-3686 (I) | rac.
7HEO | (S), (R)
<10%
AVI-4066 (I) | (S)
7HF4 | (S)
720 μM
AVI-3684 (I) | rac.
7HEM | (R), (S)
<10%
AVI-3705 (I) | rac.
7HD2 | (S)
64%
AVI-3711 (I) | rac.
7HD8 | (S)
63%
AVI-3703 (I) | rac.
7HD0 | (S)
28%
AVI-3673 (I) | rac.
7HEF | (S)
38%
AVI-3657 (I) | rac.
7HE3 | (S)
24%
AVI-3650 (I) | rac.
7HDY | (R), (S)
71%

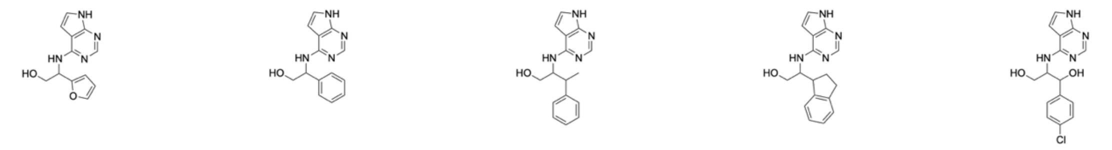

**AVI-3659** (I) | rac.<br>7HE5 | (*R*)<br>55%	**AVI-3715** (I) | rac.<br>7HDA | (*S*)<br>54%	**AVI-3669** (I) | diast.<br>7HEC | (*R*,*S*)<br>98% | 8.7 µM	**AVI-3693** (I) | diast.<br>7HET | (*R*,*R*), (*S*,*R*)<br>78%	**AVI-3718** (I) | diast.<br>7HDC | (*R*,*R*), (*S*,*R*), (*R*,*R*)<br>78%

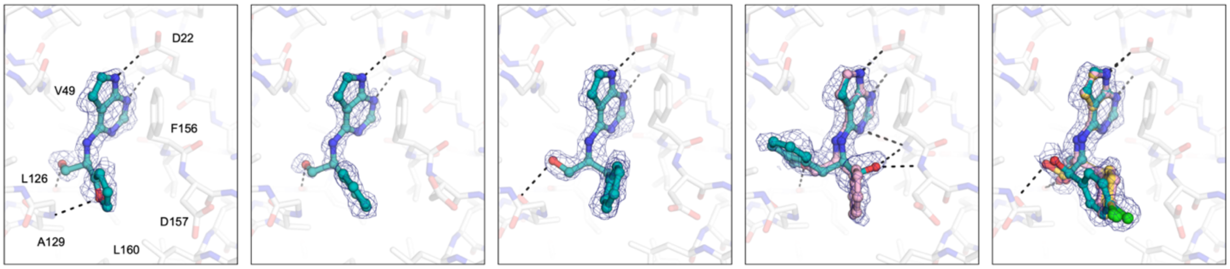

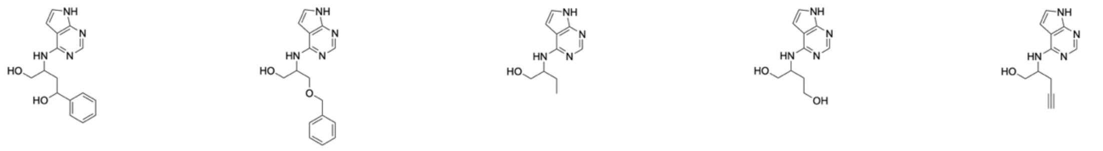

**AVI-3642** (I) | diast.<br>7HDT | (*S*,*S*)<br><10%	**AVI-3709** (I) | rac.<br>7HD5 | (*S*), (*R*)<br>29%	**AVI-3643** (I) | rac.<br>7HDU | (*R*)<br>63%	**AVI-3734** (I) | rac.<br>7HDN | (*R*)<br>86% | 52 µM	**AVI-3644** (I) | rac.<br>7HDV | (*S*)<br>43%

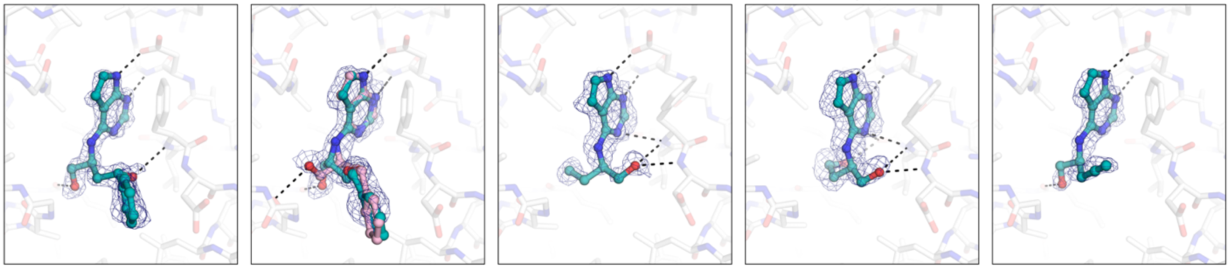

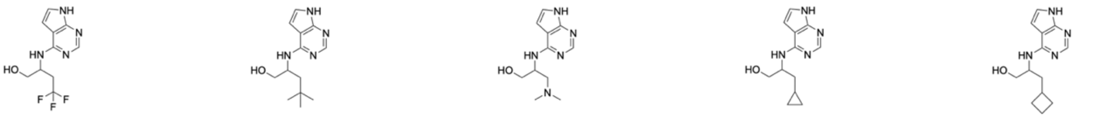

**AVI-3637** (I) | rac.<br>7HDO | (*S*)<br>50%	**AVI-3648** (I) | rac.<br>7HDX | (*S*)<br>27%	**AVI-3671** (I) | rac.<br>7HEE | (*S*)<br><10%	**AVI-3662** (I) | rac.<br>7HE7 | (*S*)<br>44%	**AVI-3641** (I) | rac.<br>7HDS | (*S*)<br>32%

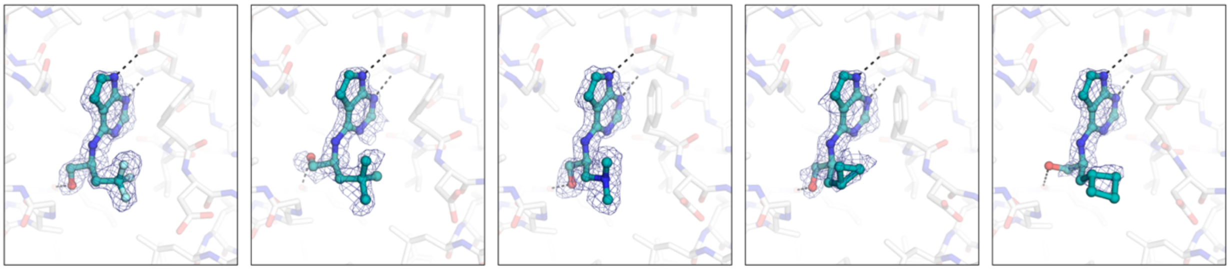

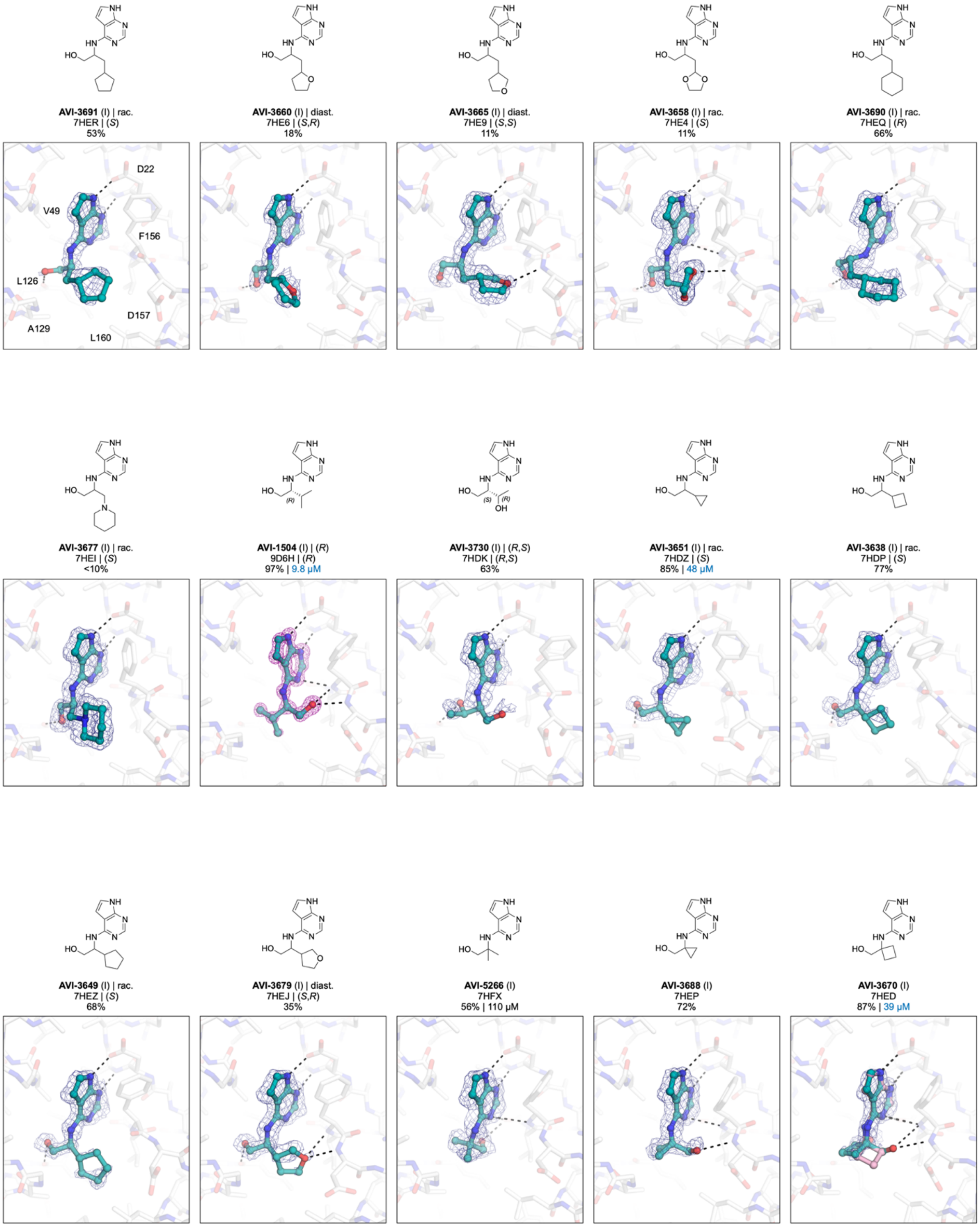

AVI-3691 (I) | rac.
7HER | (*S*)
53%
D22
V49
F156
L126
D157
A129
L160
AVI-3660 (I) | diast.
7HE6 | (*S*,*R*)
18%
AVI-3665 (I) | diast.
7HE9 | (*S*,*S*)
11%
AVI-3658 (I) | rac.
7HE4 | (*S*)
11%
AVI-3690 (I) | rac.
7HEQ | (*R*)
66%
AVI-3677 (I) | rac.
7HEI | (*S*)
<10%
AVI-1504 (I) | (*R*)
9D6H | (*R*)
97% | 9.8 µM
AVI-3730 (I) | (*R*,*S*)
7HDK | (*R*,*S*)
63%
AVI-3651 (I) | rac.
7HDZ | (*S*)
85% | 48 µM
AVI-3638 (I) | rac.
7HDP | (*S*)
77%
AVI-3649 (I) | rac.
7HEZ | (*S*)
68%
AVI-3679 (I) | diast.
7HEJ | (*S*,*R*)
35%
AVI-5266 (I)
7HFX
56% | 110 µM
AVI-3688 (I)
7HEP
72%
AVI-3670 (I)
7HED
87% | 39 µM

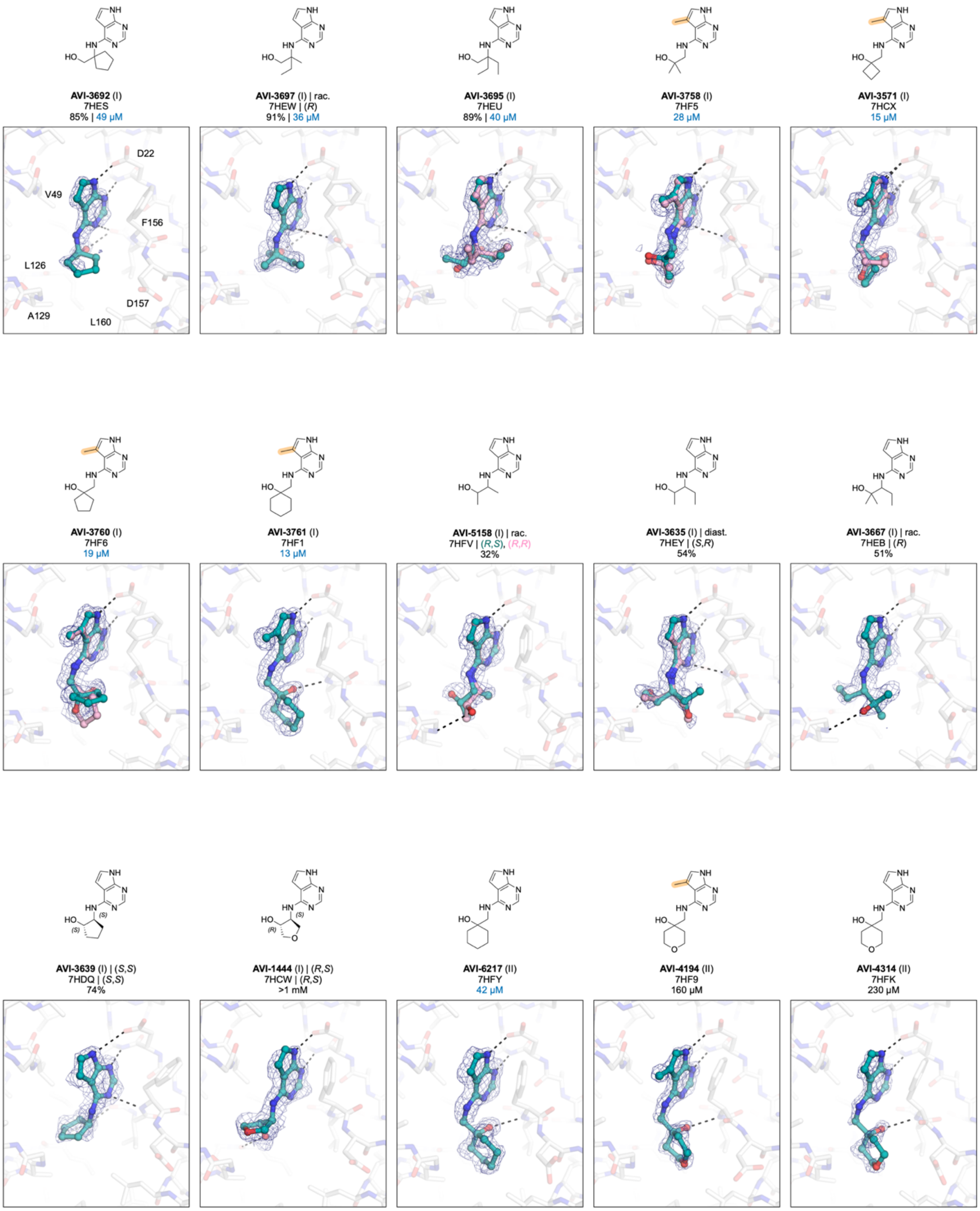

AVI-3692 (I)
7HES
85% | 49 μM
D22
V49
F156
L126
D157
A129
L160
AVI-3697 (I) | rac.
7HEW | (*R*)
91% | 36 μM
AVI-3695 (I)
7HEU
89% | 40 μM
AVI-3758 (I)
7HF5
28 μM
AVI-3571 (I)
7HCX
15 μM
AVI-3760 (I)
7HF6
19 μM
AVI-3761 (I)
7HF1
13 μM
AVI-5158 (I) | rac.
7HFV | (*R*,*S*), (*R*,*R*)
32%
AVI-3635 (I) | diast.
7HEY | (*S*,*R*)
54%
AVI-3667 (I) | rac.
7HEB | (*R*)
51%
AVI-3639 (I) | (*S*,*S*)
7HDQ | (*S*,*S*)
74%
AVI-1444 (I) | (*R*,*S*)
7HCW | (*R*,*S*)
>1 mM
AVI-6217 (II)
7HFY
42 μM
AVI-4194 (II)
7HF9
160 μM
AVI-4314 (II)
7HFK
230 μM

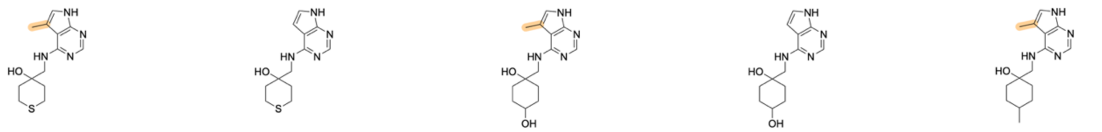

AVI-4195 (II)
7HFA
52 μM

AVI-4319 (II)
7HFL
170 μM

AVI-4329 (II) | diast.
7HF8 | (*r*,*r*)
>1 mM

AVI-6220 (II) | diast.
7HFZ | (*r*,*r*)
260 μM

AVI-4331 (II) | diast.
7HFB | (*r*,*r*)
210 μM

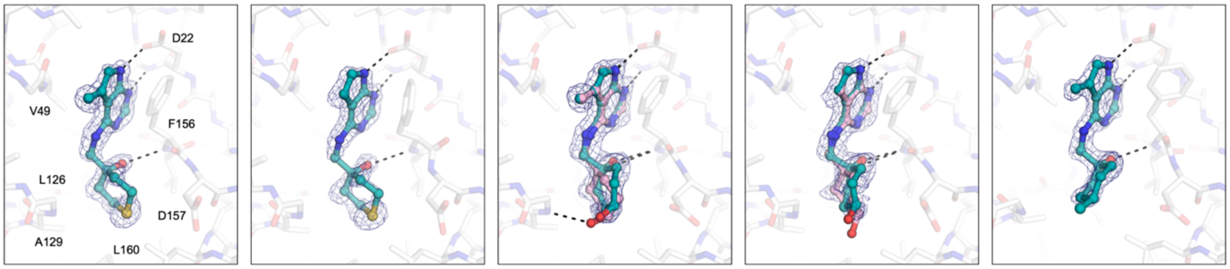

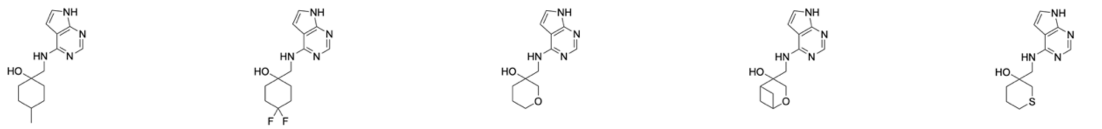

AVI-4312 (II) | diast.
7HFI | (*r*,*r*)
21 μM

AVI-4308 (II)
7HFG
140 μM

AVI-4311 (II) | rac.
7HFH | (*R*)
170 μM

AVI-4313 (II) | rac..
7HFJ | (*S*)
130 μM

AVI-4322 (II) | rac.
7HFN | (*R*)
53 μM

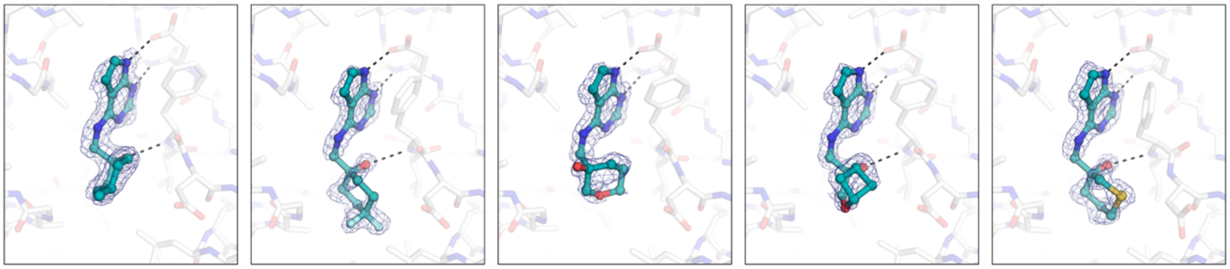

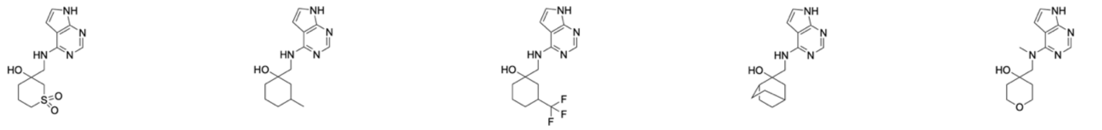

AVI-4317 (II) | rac.
9D6I | (*R*)
110 μM

AVI-4307 (II) | diast.
7HFF | (*R*,*S*)
40 μM

AVI-4309 (II) | diast.
7HFO | (*S*,*R*)
150 μM

AVI-4320 (II) | rac.
7HFM | (*R*)
83 μM

AVI-4318 (II)
7HFP
71 μM

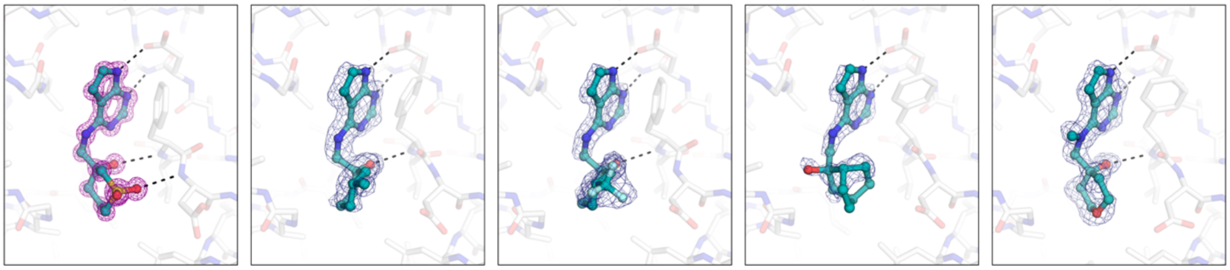

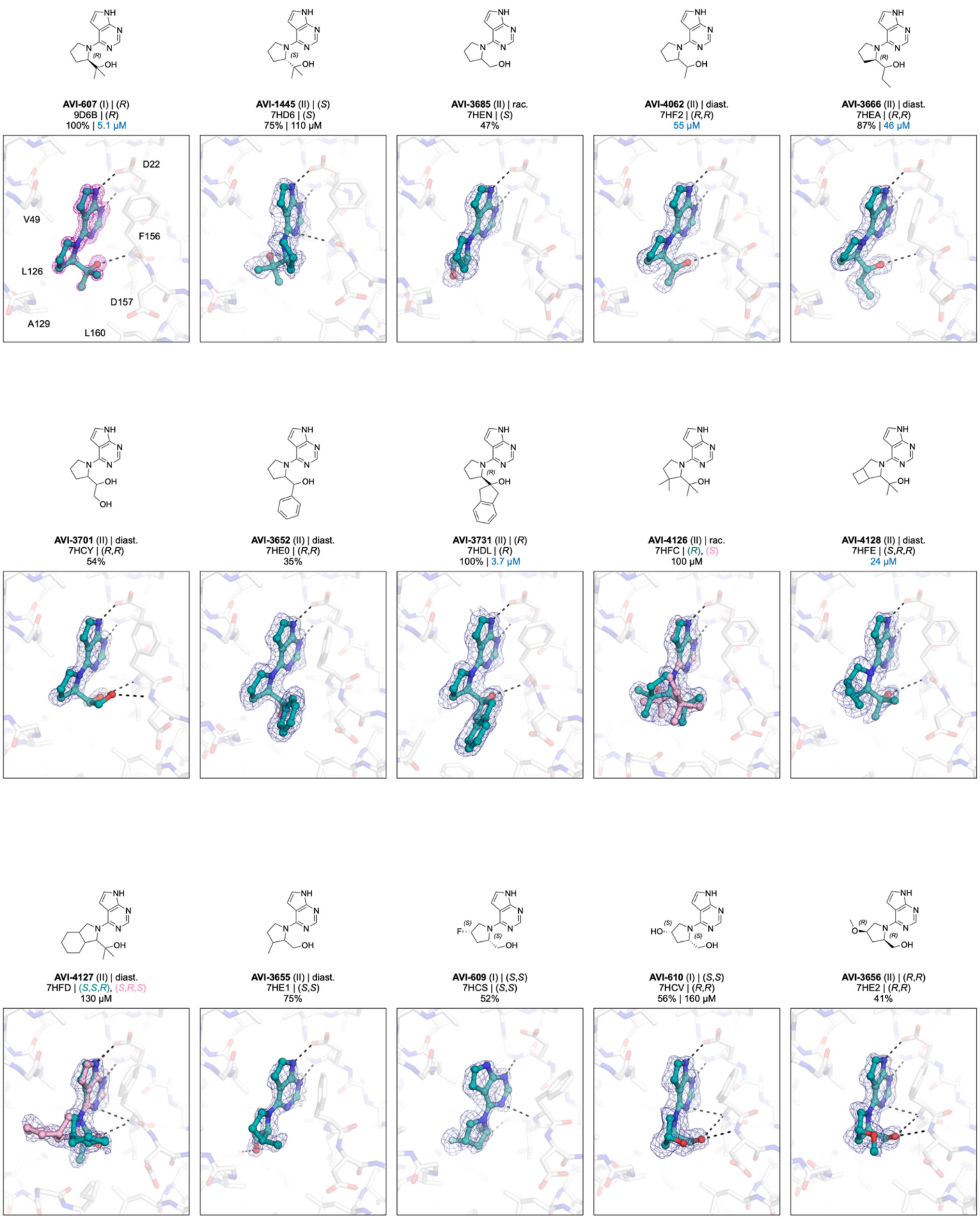

AVI-607 (I) | (R)
9D6B | (R)
100% | 5.1 µM
D22
V49
F156
L126
D157
A129
L160
AVI-1445 (II) | (S)
7HD6 | (S)
75% | 110 µM
AVI-3685 (II) | rac.
7HEN | (S)
47%
AVI-4062 (II) | diast.
7HF2 | (R,R)
55 µM
AVI-3666 (II) | diast.
7HEA | (R,R)
87% | 46 µM
AVI-3701 (II) | diast.
7HCY | (R,R)
54%
AVI-3652 (II) | diast.
7HE0 | (R,R)
35%
AVI-3731 (II) | (R)
7HDL | (R)
100% | 3.7 µM
AVI-4126 (II) | rac.
7HFC | (R), (S)
100 µM
AVI-4128 (II) | diast.
7HFE | (S,R,R)
24 µM
AVI-4127 (II) | diast.
7HFD | (S,S,R), (S,R,S)
130 µM
AVI-3655 (II) | diast.
7HE1 | (S,S)
75%
AVI-609 (I) | (S,S)
7HCS | (S,S)
52%
AVI-610 (I) | (S,S)
7HCV | (R,R)
56% | 160 µM
AVI-3656 (II) | (R,R)
7HE2 | (R,R)
41%

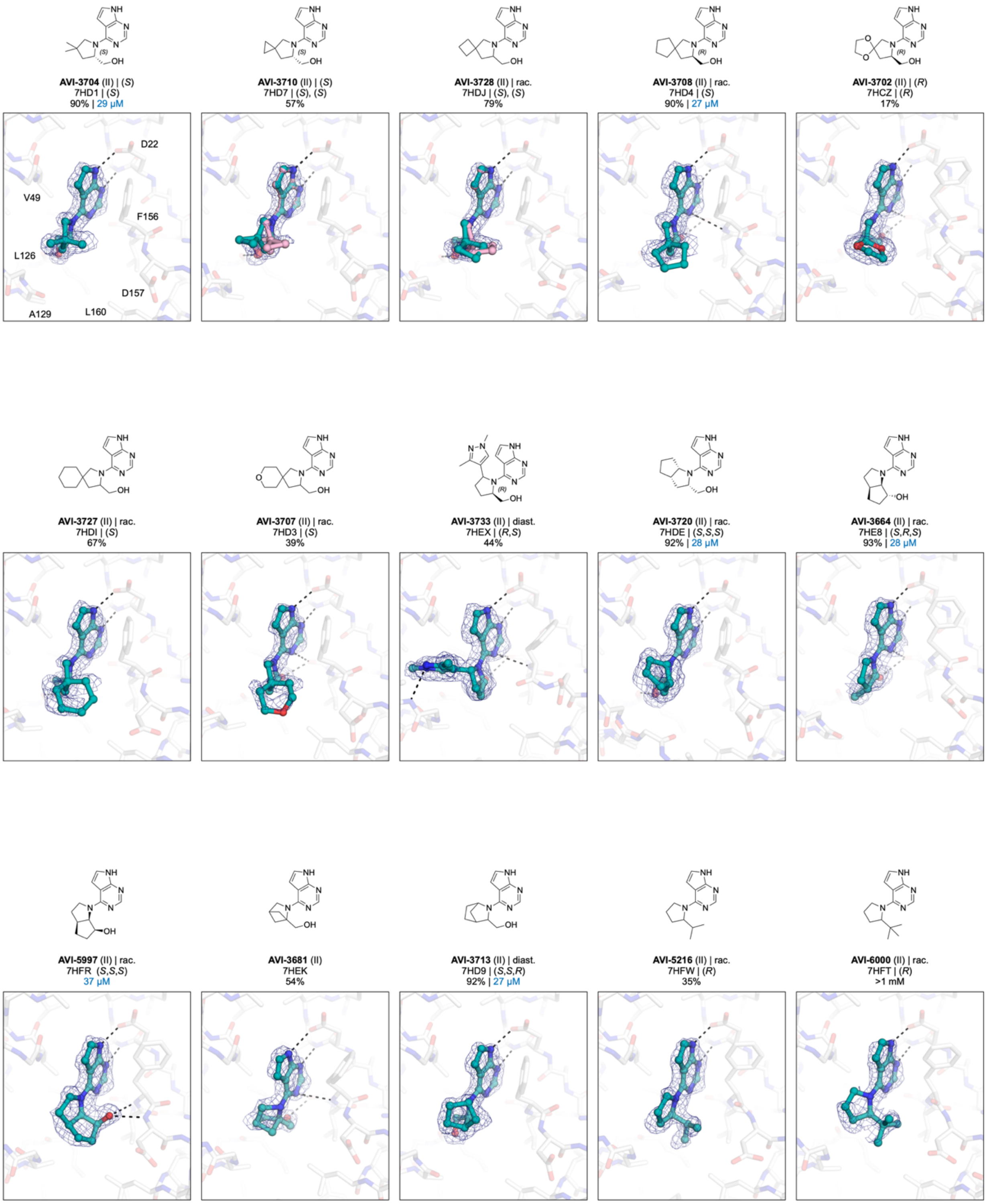

AVI-3704 (II) | (S)
7HD1 | (S)
90% | 29 µM
AVI-3710 (II) | (S)
7HD7 | (S), (S)
57%
AVI-3728 (II) | rac.
7HDJ | (S), (S)
79%
AVI-3708 (II) | rac.
7HD4 | (S)
90% | 27 µM
AVI-3702 (II) | (R)
7HCZ | (R)
17%
D22
V49
F156
L126
D157
A129
L160
AVI-3727 (II) | rac.
7HDI | (S)
67%
AVI-3707 (II) | rac.
7HD3 | (S)
39%
AVI-3733 (II) | diast.
7HEX | (R,S)
44%
AVI-3720 (II) | rac.
7HDE | (S,S,S)
92% | 28 µM
AVI-3664 (II) | rac.
7HE8 | (S,R,S)
93% | 28 µM
AVI-5997 (II) | rac.
7HFR (S,S,S)
37 µM
AVI-3681 (II)
7HEK
54%
AVI-3713 (II) | diast.
7HD9 | (S,S,R)
92% | 27 µM
AVI-5216 (II) | rac.
7HFW | (R)
35%
AVI-6000 (II) | rac.
7HFT | (R)
>1 mM

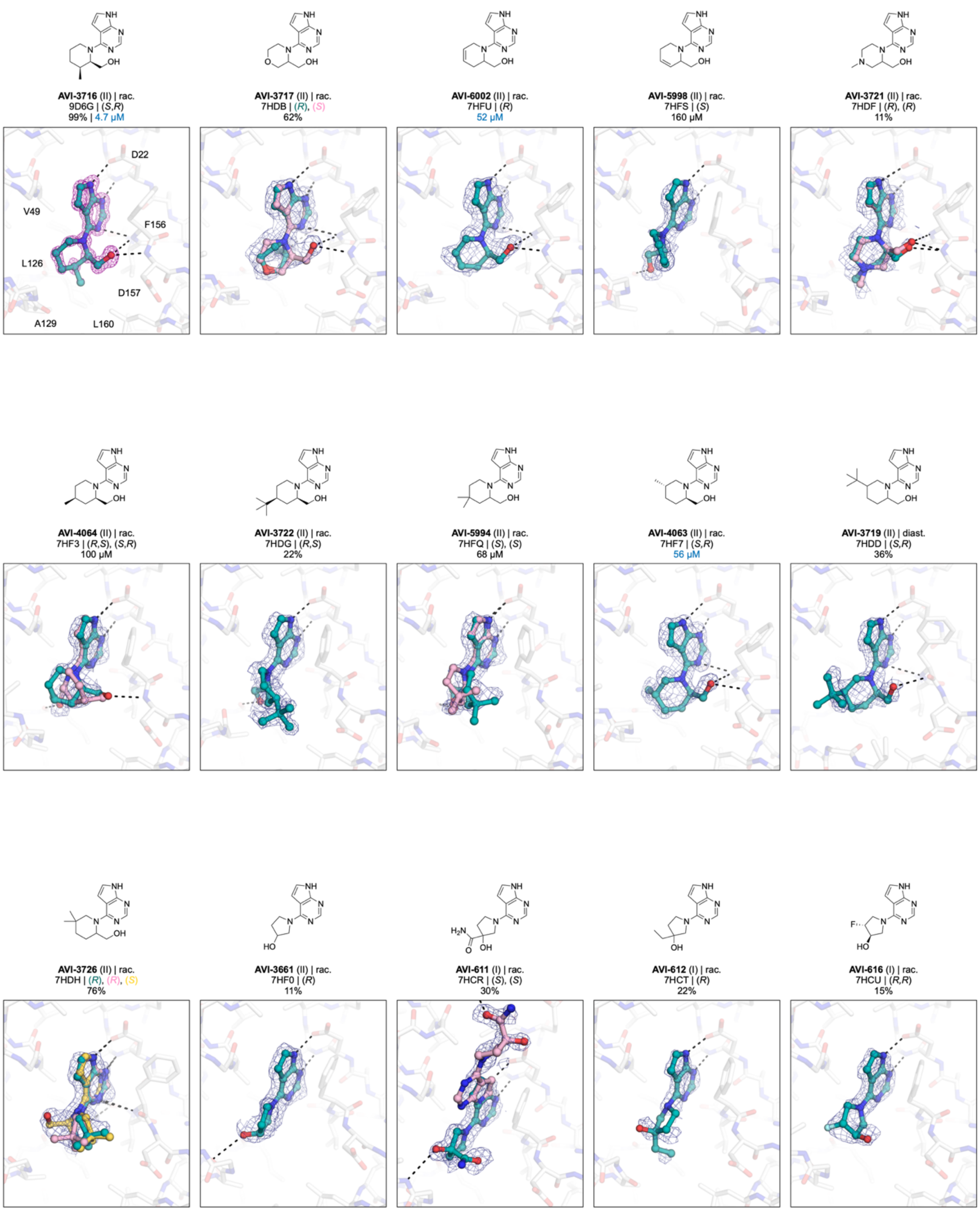
AVI-3716 (II) | rac.
9D6G | (S,R)
99% | 4.7 μM
D22
V49
F156
L126
D157
A129
L160
AVI-3717 (II) | rac.
7HDB | (R), (S)
62%
AVI-6002 (II) | rac.
7HFU | (R)
52 μM
AVI-5998 (II) | rac.
7HFS | (S)
160 μM
AVI-3721 (II) | rac.
7HDF | (R), (R)
11%
AVI-4064 (II) | rac.
7HF3 | (R,S), (S,R)
100 μM
AVI-3722 (II) | rac.
7HDG | (R,S)
22%
AVI-5994 (II) | rac.
7HFQ | (S), (S)
68 μM
AVI-4063 (II) | rac.
7HF7 | (S,R)
56 μM
AVI-3719 (II) | diast.
7HDD | (S,R)
36%
AVI-3726 (II) | rac.
7HDH | (R), (R), (S)
76%
AVI-3661 (II) | rac.
7HF0 | (R)
11%
AVI-611 (I) | rac.
7HCR | (S), (S)
30%
AVI-612 (I) | rac.
7HCT | (R)
22%
AVI-616 (I) | rac.
7HCU | (R,R)
15%

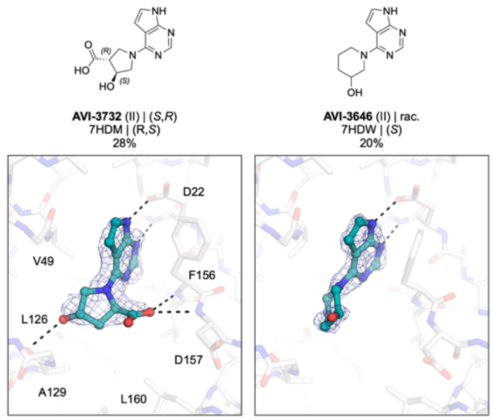

Fig. S2.

Chemical structures and electron density maps for the 137 X-ray crystal structures reported in this work. PanDDA event maps (blue mesh, 2 σ) or mF_O-DF_C difference electron density maps prior to ligand placement (purple mesh, 5 σ) are contoured around ligands. Compounds were synthesized as pure isomers, as racamates (rac.) or as mixtures of diastereomers (diast.) and the stereochemistry of the modeled compound is indicated. Results for the preliminary HTRF screen (% competition at 200 µM, mean of three technical replicates) and the confirmatory dose-response screen (IC_{50}, best fit value of 3-4 technical replicates) are indicated.

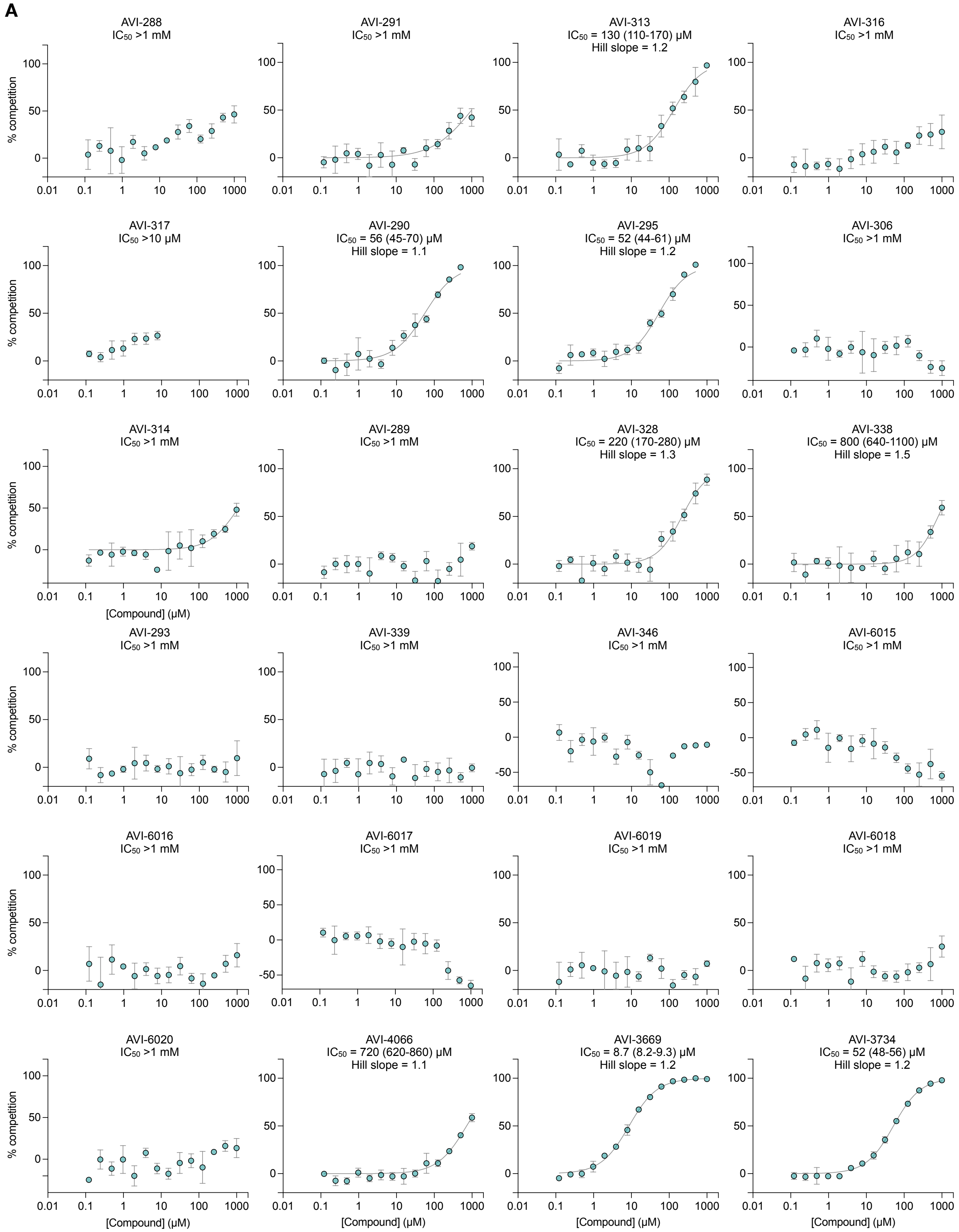
A
AVI-288
IC50 >1 mM
AVI-291
IC50 >1 mM
AVI-313
IC50 = 130 (110-170) μM
Hill slope = 1.2
AVI-316
IC50 >1 mM
AVI-317
IC50 >10 μM
AVI-290
IC50 = 56 (45-70) μM
Hill slope = 1.1
AVI-295
IC50 = 52 (44-61) μM
Hill slope = 1.2
AVI-306
IC50 >1 mM
AVI-314
IC50 >1 mM
AVI-289
IC50 >1 mM
AVI-328
IC50 = 220 (170-280) μM
Hill slope = 1.3
AVI-338
IC50 = 800 (640-1100) μM
Hill slope = 1.5
AVI-293
IC50 >1 mM
AVI-339
IC50 >1 mM
AVI-346
IC50 >1 mM
AVI-6015
IC50 >1 mM
AVI-6016
IC50 >1 mM
AVI-6017
IC50 >1 mM
AVI-6019
IC50 >1 mM
AVI-6018
IC50 >1 mM
AVI-6020
IC50 >1 mM
AVI-4066
IC50 = 720 (620-860) μM
Hill slope = 1.1
AVI-3669
IC50 = 8.7 (8.2-9.3) μM
Hill slope = 1.2
AVI-3734
IC50 = 52 (48-56) μM
Hill slope = 1.2
% competition
[Compound] (μM)

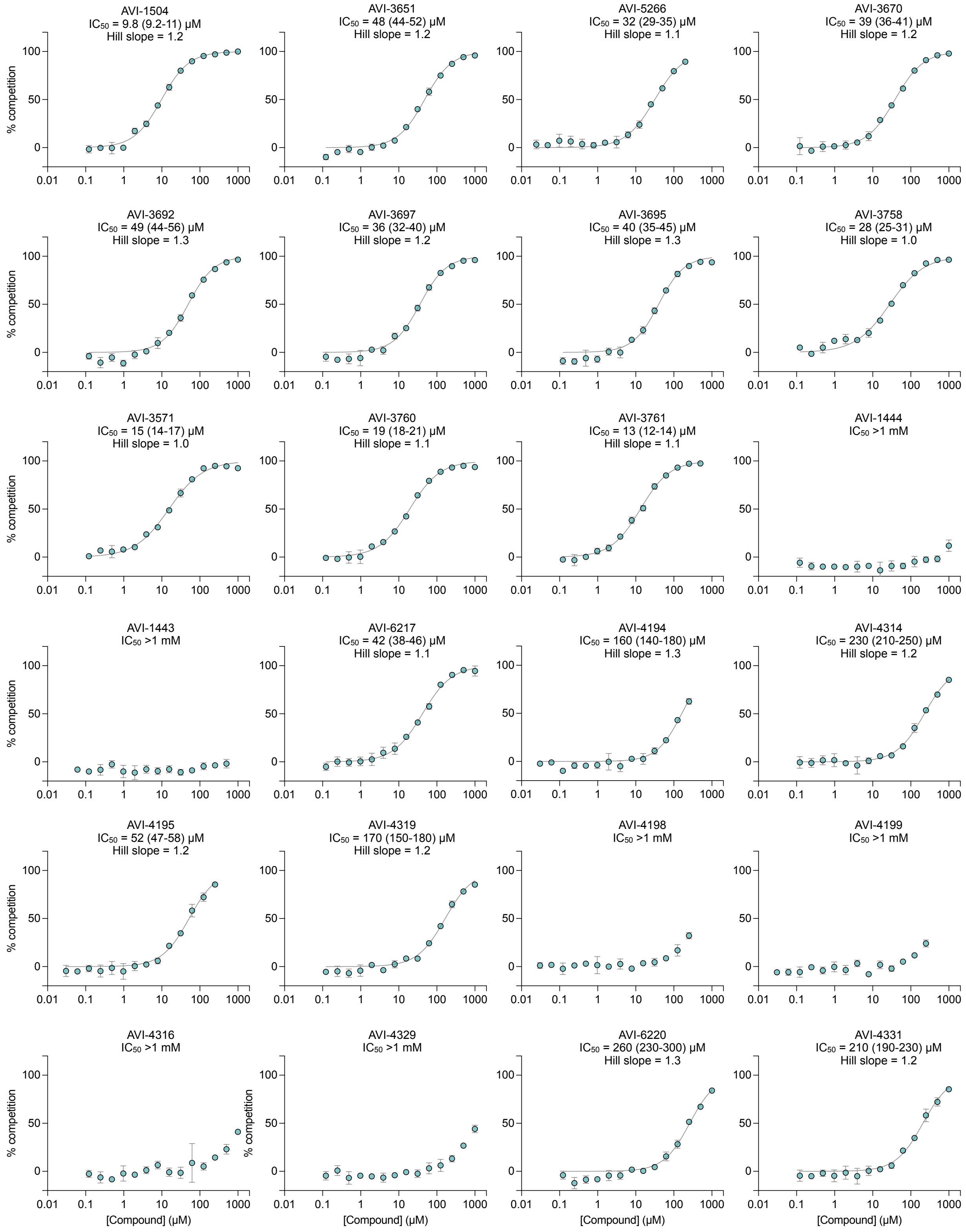

AVI-1504
IC50 = 9.8 (9.2-11) μM
Hill slope = 1.2
AVI-3651
IC50 = 48 (44-52) μM
Hill slope = 1.2
AVI-5266
IC50 = 32 (29-35) μM
Hill slope = 1.1
AVI-3670
IC50 = 39 (36-41) μM
Hill slope = 1.2
AVI-3692
IC50 = 49 (44-56) μM
Hill slope = 1.3
AVI-3697
IC50 = 36 (32-40) μM
Hill slope = 1.2
AVI-3695
IC50 = 40 (35-45) μM
Hill slope = 1.3
AVI-3758
IC50 = 28 (25-31) μM
Hill slope = 1.0
AVI-3571
IC50 = 15 (14-17) μM
Hill slope = 1.0
AVI-3760
IC50 = 19 (18-21) μM
Hill slope = 1.1
AVI-3761
IC50 = 13 (12-14) μM
Hill slope = 1.1
AVI-1444
IC50 >1 mM
AVI-1443
IC50 >1 mM
AVI-6217
IC50 = 42 (38-46) μM
Hill slope = 1.1
AVI-4194
IC50 = 160 (140-180) μM
Hill slope = 1.3
AVI-4314
IC50 = 230 (210-250) μM
Hill slope = 1.2
AVI-4195
IC50 = 52 (47-58) μM
Hill slope = 1.2
AVI-4319
IC50 = 170 (150-180) μM
Hill slope = 1.2
AVI-4198
IC50 >1 mM
AVI-4199
IC50 >1 mM
AVI-4316
IC50 >1 mM
AVI-4329
IC50 >1 mM
AVI-6220
IC50 = 260 (230-300) μM
Hill slope = 1.3
AVI-4331
IC50 = 210 (190-230) μM
Hill slope = 1.2
% competition
[Compound] (μM)

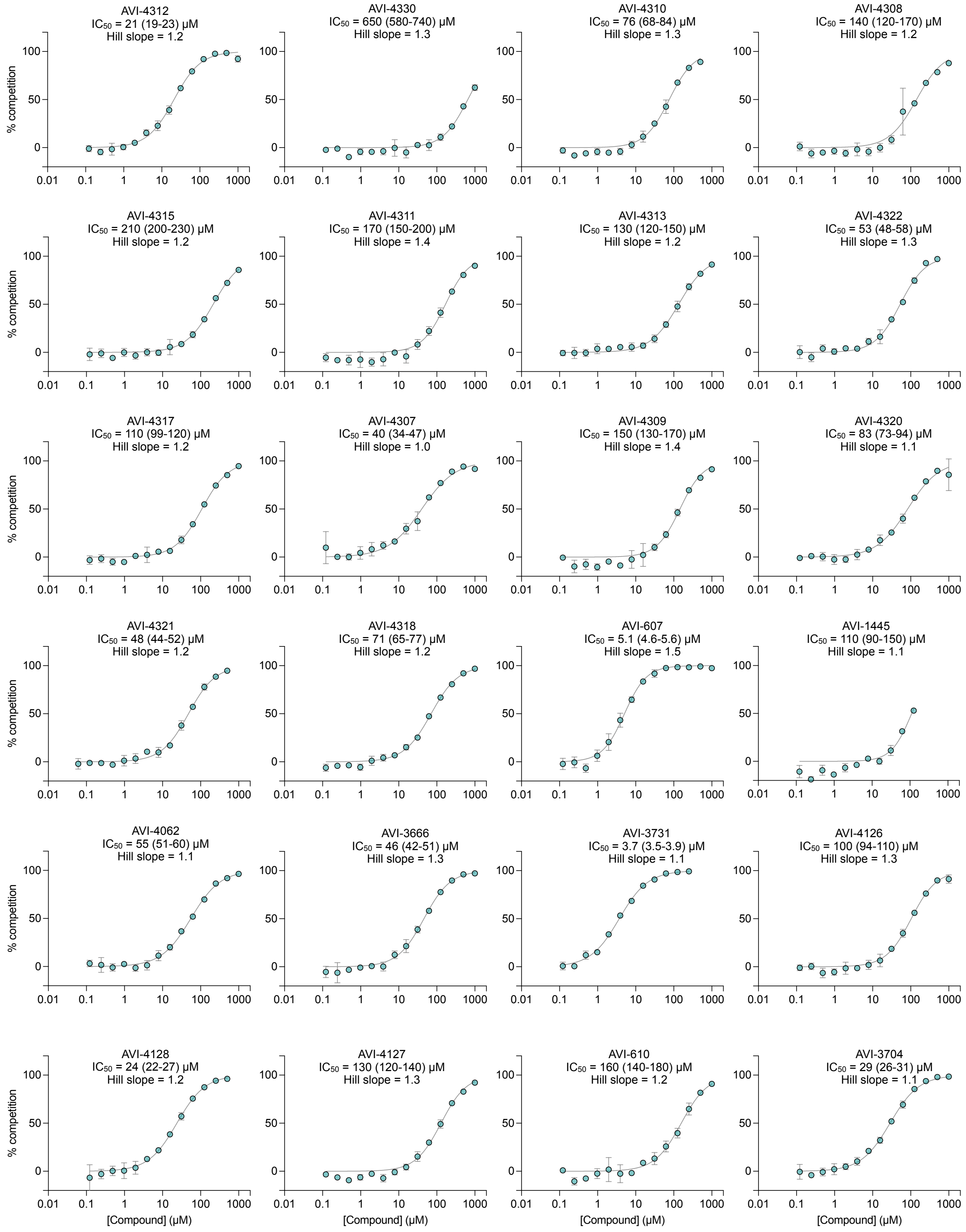
AVI-4312
IC50 = 21 (19-23) μM
Hill slope = 1.2
AVI-4330
IC50 = 650 (580-740) μM
Hill slope = 1.3
AVI-4310
IC50 = 76 (68-84) μM
Hill slope = 1.3
AVI-4308
IC50 = 140 (120-170) μM
Hill slope = 1.2
AVI-4315
IC50 = 210 (200-230) μM
Hill slope = 1.2
AVI-4311
IC50 = 170 (150-200) μM
Hill slope = 1.4
AVI-4313
IC50 = 130 (120-150) μM
Hill slope = 1.2
AVI-4322
IC50 = 53 (48-58) μM
Hill slope = 1.3
AVI-4317
IC50 = 110 (99-120) μM
Hill slope = 1.2
AVI-4307
IC50 = 40 (34-47) μM
Hill slope = 1.0
AVI-4309
IC50 = 150 (130-170) μM
Hill slope = 1.4
AVI-4320
IC50 = 83 (73-94) μM
Hill slope = 1.1
AVI-4321
IC50 = 48 (44-52) μM
Hill slope = 1.2
AVI-4318
IC50 = 71 (65-77) μM
Hill slope = 1.2
AVI-607
IC50 = 5.1 (4.6-5.6) μM
Hill slope = 1.5
AVI-1445
IC50 = 110 (90-150) μM
Hill slope = 1.1
AVI-4062
IC50 = 55 (51-60) μM
Hill slope = 1.1
AVI-3666
IC50 = 46 (42-51) μM
Hill slope = 1.3
AVI-3731
IC50 = 3.7 (3.5-3.9) μM
Hill slope = 1.1
AVI-4126
IC50 = 100 (94-110) μM
Hill slope = 1.3
AVI-4128
IC50 = 24 (22-27) μM
Hill slope = 1.2
AVI-4127
IC50 = 130 (120-140) μM
Hill slope = 1.3
AVI-610
IC50 = 160 (140-180) μM
Hill slope = 1.2
AVI-3704
IC50 = 29 (26-31) μM
Hill slope = 1.1
% competition
[Compound] (μM)

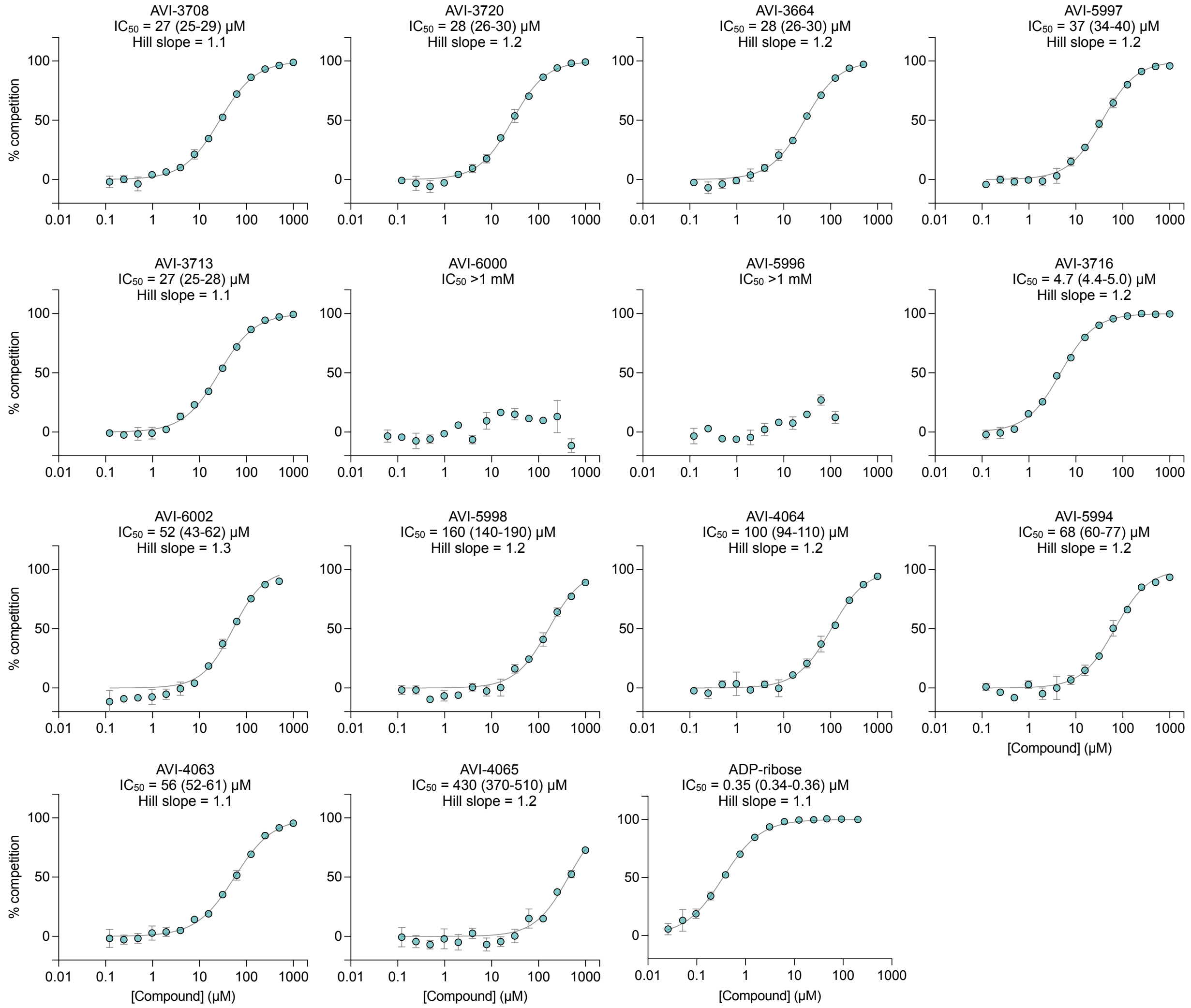

AVI-3708
IC_{50} = 27 (25-29) μM
Hill slope = 1.1
AVI-3720
IC_{50} = 28 (26-30) μM
Hill slope = 1.2
AVI-3664
IC_{50} = 28 (26-30) μM
Hill slope = 1.2
AVI-5997
IC_{50} = 37 (34-40) μM
Hill slope = 1.2
AVI-3713
IC_{50} = 27 (25-28) μM
Hill slope = 1.1
AVI-6000
IC_{50} >1 mM
AVI-5996
IC_{50} >1 mM
AVI-3716
IC_{50} = 4.7 (4.4-5.0) μM
Hill slope = 1.2
AVI-6002
IC_{50} = 52 (43-62) μM
Hill slope = 1.3
AVI-5998
IC_{50} = 160 (140-190) μM
Hill slope = 1.2
AVI-4064
IC_{50} = 100 (94-110) μM
Hill slope = 1.2
AVI-5994
IC_{50} = 68 (60-77) μM
Hill slope = 1.2
AVI-4063
IC_{50} = 56 (52-61) μM
Hill slope = 1.1
AVI-4065
IC_{50} = 430 (370-510) μM
Hill slope = 1.2
ADP-ribose
IC_{50} = 0.35 (0.34-0.36) μM
Hill slope = 1.1
% competition
[Compound] (μM)

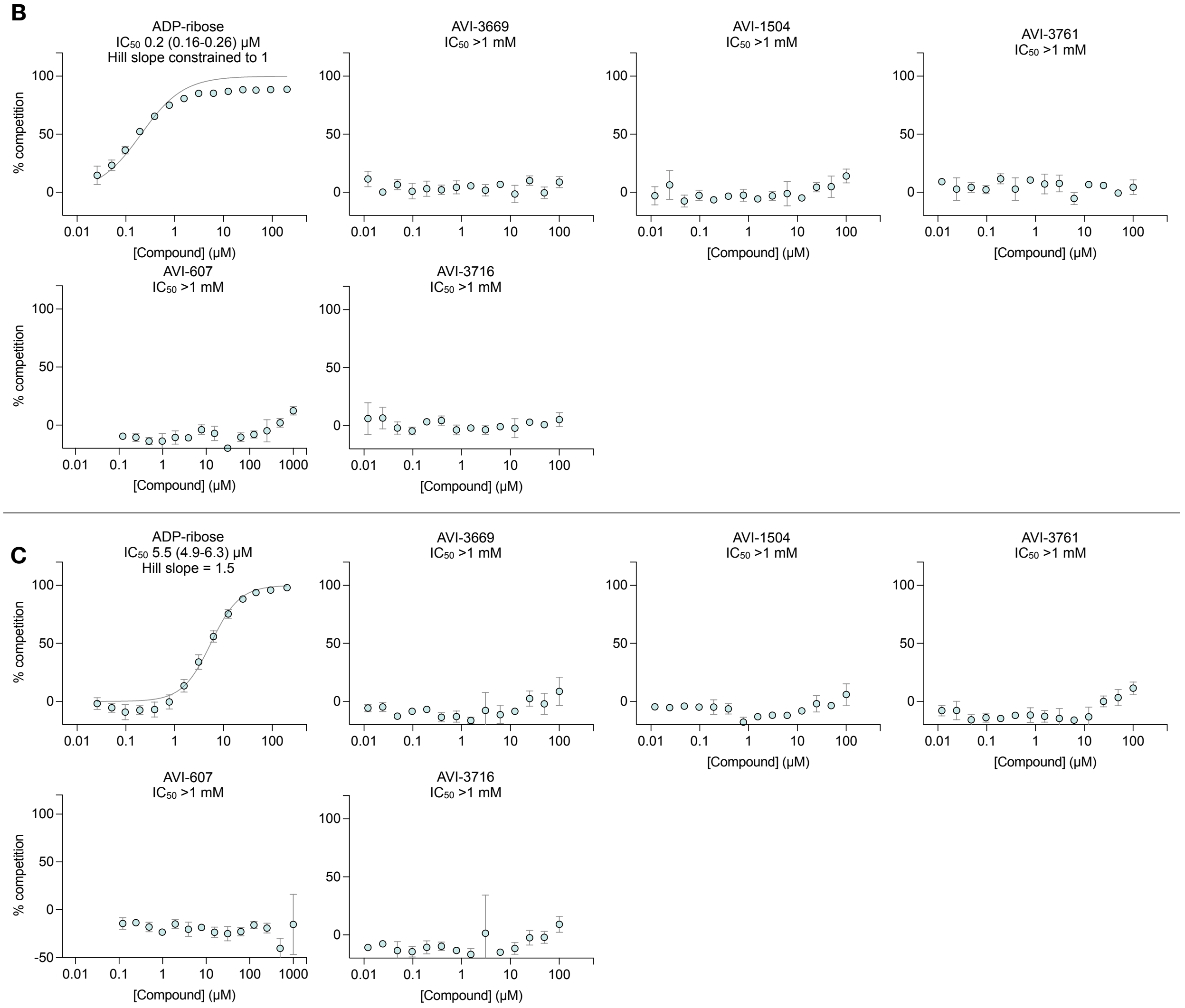

Fig. S3.

HTRF binding curves for all compounds tested. Curves are shown for SARS-Cov-2 Mac1 (A) and the human macrodomains MacroD2 (**B**) and Targ1 (**C**). Data points are mean ± SD for 3-4 technical replicates at each compound concentration, except for ADP-ribose with Mac1, where there were 12 technical replicates. The IC_{50} value was determined by non-linear regression of a four-parameter sigmoidal dose-response equation in GraphPad Prism (version 10.1.1). The best fit value is indicated along with the 95% confidence interval and the Hill slope. The Hill slope was constrained to 1 for the MacroD2 ADP-ribose binding curve.

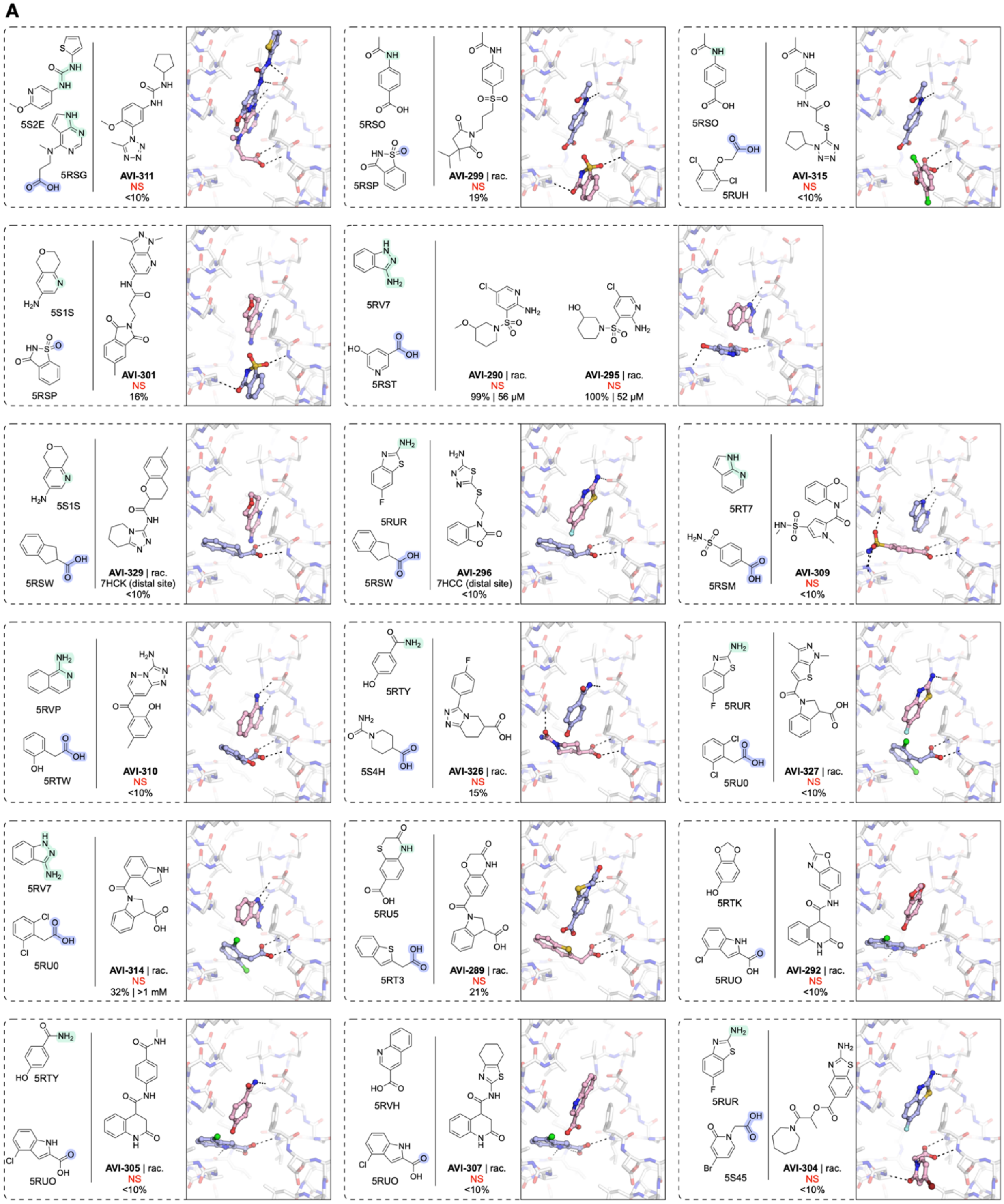
A
5S2E
5RSG
AVI-311
NS
<10%
5RSO
5RSP
AVI-299 | rac.
NS
19%
5RSO
5RUH
AVI-315
NS
<10%
5S1S
5RSP
AVI-301
NS
16%
5RV7
5RST
AVI-290 | rac.
NS
99% | 56 µM
AVI-295 | rac.
NS
100% | 52 µM
5S1S
5RSW
AVI-329 | rac.
7HCK (distal site)
<10%
5RUR
5RSW
AVI-296
7HCC (distal site)
<10%
5RT7
5RSM
AVI-309
NS
<10%
5RVP
5RTW
AVI-310
NS
<10%
5RTY
5S4H
AVI-326 | rac.
NS
15%
5RUR
5RU0
AVI-327 | rac.
NS
<10%
5RV7
5RU0
AVI-314 | rac.
NS
32% | >1 mM
5RU5
5RT3
AVI-289 | rac.
NS
21%
5RTK
5RUO
AVI-292 | rac.
NS
<10%
5RTY
5RUO
AVI-305 | rac.
NS
<10%
5RVH
5RUO
AVI-307 | rac.
NS
<10%
5RUR
5S45
AVI-304 | rac.
NS
<10%

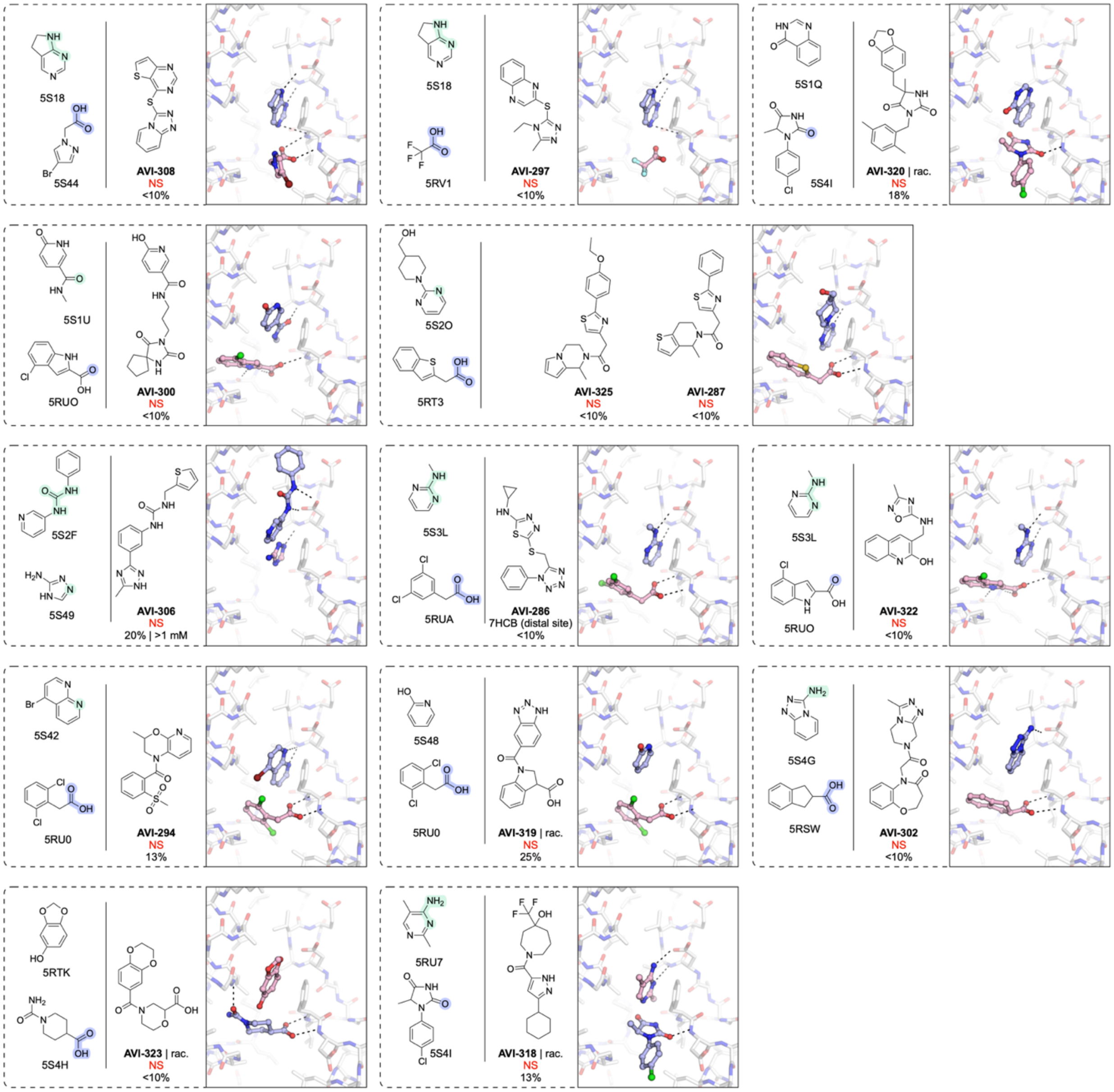
5S18
5S44
AVI-308
NS
<10%
5S18
5RV1
AVI-297
NS
<10%
5S1Q
5S4I
AVI-320 | rac.
NS
18%
5S1U
5RUO
AVI-300
NS
<10%
5S2O
5RT3
AVI-325
NS
<10%
AVI-287
NS
<10%
5S2F
5S49
AVI-306
NS
20% | >1 mM
5S3L
5RUA
AVI-286
7HCB (distal site)
<10%
5S3L
5RUO
AVI-322
NS
<10%
5S42
5RU0
AVI-294
NS
13%
5S48
5RU0
AVI-319 | rac.
NS
25%
5S4G
5RSW
AVI-302
NS
<10%
5RTK
5S4H
AVI-323 | rac.
NS
<10%
5RU7
5S4I
AVI-318 | rac.
NS
13%

B

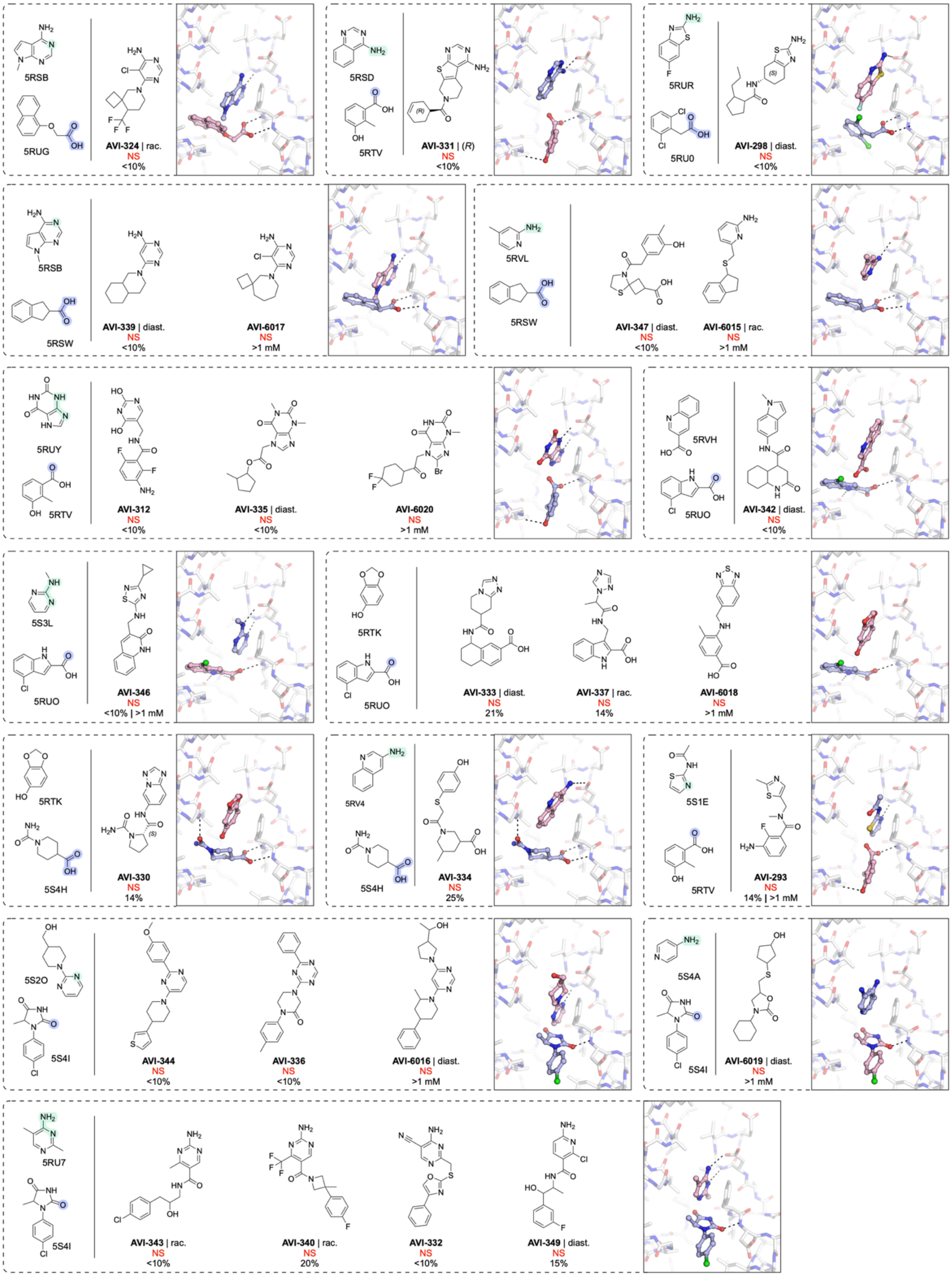
5RSB
5RUG
AVI-324 | rac.
NS
<10%
5RSD
5RTV
AVI-331 | (R)
NS
<10%
5RUR
5RU0
AVI-298 | diast.
NS
<10%
5RSB
5RSW
AVI-339 | diast.
NS
<10%
AVI-6017
NS
>1 mM
5RVL
5RSW
AVI-347 | diast.
NS
<10%
AVI-6015 | rac.
NS
>1 mM
5RUY
5RTV
AVI-312
NS
<10%
AVI-335 | diast.
NS
<10%
AVI-6020
NS
>1 mM
5RVH
5RUO
AVI-342 | diast.
NS
<10%
5S3L
5RUO
AVI-346
NS
<10% | >1 mM
5RTK
5RUO
AVI-333 | diast.
NS
21%
AVI-337 | rac.
NS
14%
AVI-6018
NS
>1 mM
5RTK
5S4H
AVI-330
NS
14%
5RV4
5S4H
AVI-334
NS
25%
5S1E
5RTV
AVI-293
NS
14% | >1 mM
5S2O
5S4I
AVI-344
NS
<10%
AVI-336
NS
<10%
AVI-6016 | diast.
NS
>1 mM
5S4A
5S4I
AVI-6019 | diast.
NS
>1 mM
5RU7
5S4I
AVI-343 | rac.
NS
<10%
AVI-340 | rac.
NS
20%
AVI-332
NS
<10%
AVI-349 | diast.
NS
15%

Fig. S4.

Compounds synthesized from the FrankenROCS (A) and the TS-FrankenROCS (B) pipelines where no X-ray crystal structure was determined. Compounds were purchased as pure isomers, as racamates (rac.) or mixtures of diastereomers (diast.). The X-ray crystal structures of the parent fragments (transparent blue/pink sticks) are shown. The fragment atoms contacting the adenine and oxyanion subsites are shaded green and blue respectively. Hydrogen bonds between the fragment and Mac1 are shown as dashed black lines. Results for the preliminary HTRF screen (% competition at 200 μM, mean of three technical replicates) and the confirmatory dose-response screen (IC_{50}, best fit value of 3-4 technical replicates) are indicated.

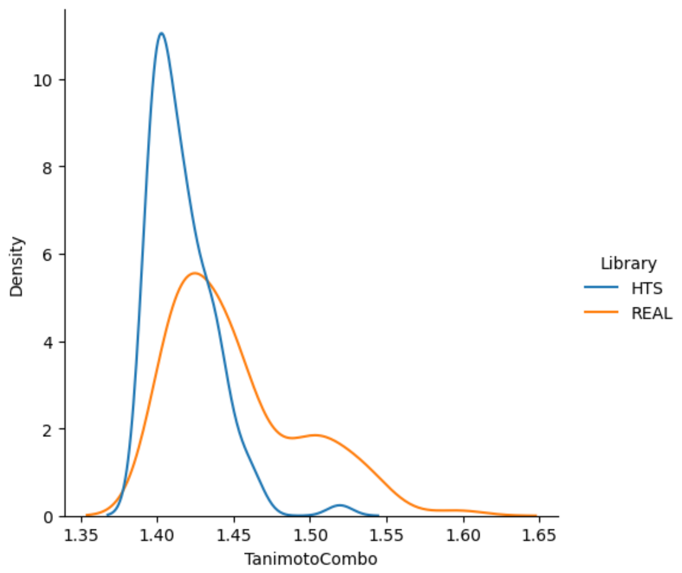

Fig. S5.
Comparison of the top 100 TanimotoCombo scores for the Enamine HTS collection versus the Enamine REAL database.

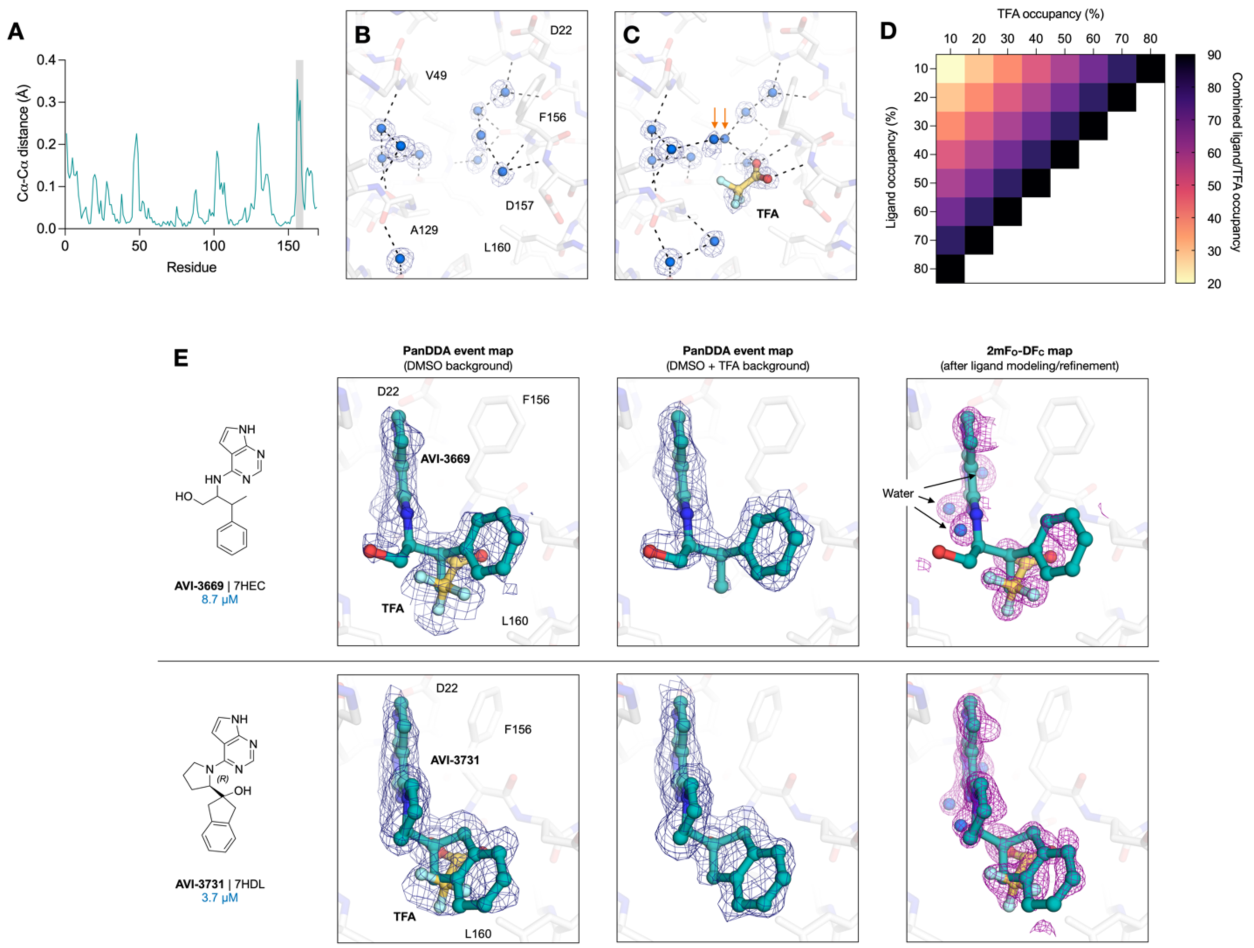

Fig. S6.

TFA subtraction improves the PanDDA map interpretability. (**A**) Pairwise Cα-Cα distances between the DMSO- and TFA-reference structures. Residues 155-160 are shaded. These were modeled as an alternative state in the TFA-bound multi-state models. (**B**) Average electron density map calculated by PanDDA from 34 datasets collected from DMSO-soaked crystals with the DMSO reference structure shown (UCSF-P0075) (blue mesh contoured at 0.8 σ around selected water molecules (blue spheres)). Hydrogen bond networks with water molecules are shown with dashed black lines. (**C**) Average electron density map calculated by PanDDA from 44 datasets collected from TFA/DMSO-soaked crystals with the TFA reference structure shown (UCSF-P3816) (blue mesh contoured at 0.8 σ around selected water molecules (blue spheres)). The two water molecules included in the TFA-bound state of the three-state multi-conformer model (DMSO-only, TFA-bound, ligand-bound) are indicated with orange arrows. Although some evidence for these waters was observed in the DMSO-soaked background map, they were only included in the TFA reference structure. (**D**) Heat map showing the grid search performed to determine ligand/TFA occupancy. Each square represents a separate refinement run where the ligand/TFA occupancies were set to the indicated values. (**E**) Examples of ligands where the TFA density was removed in the PanDDA event maps (blue mesh, 2 σ) using the TFA/DMSO-background map. The $2mF_O$-DF_C map (purple mesh, 1 σ) calculated with the final refined model are contoured around the ligands/TFA and overlapping water molecules.

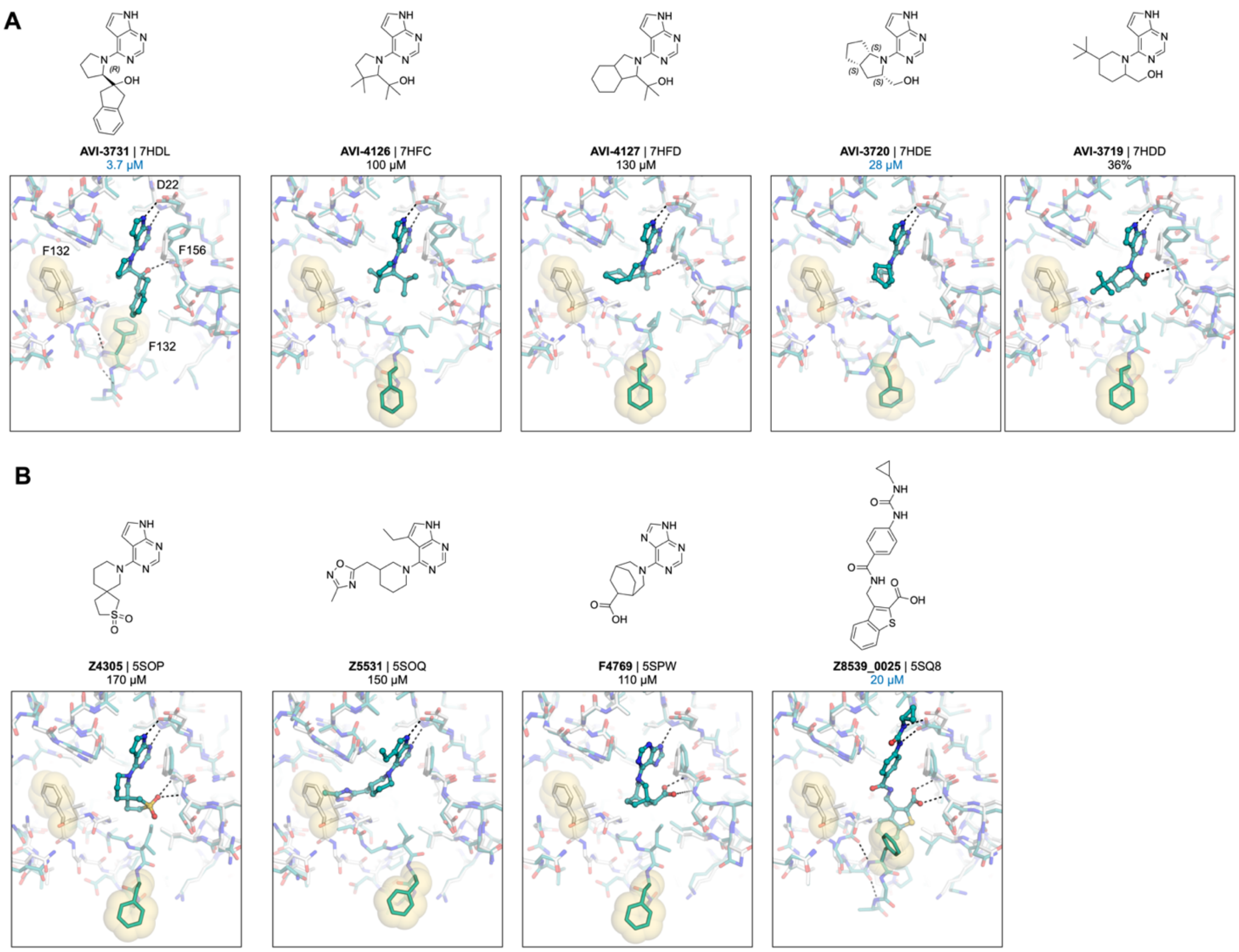

Fig. S7.

AVI-313 analogues stabilize an alternative, open state of the Phe132 loop. (**A**) Top: Chemical structures of AVI-131 ligands that stabilize the open state of Mac1 reported in this work. Bottom: alignment of the apo X-ray crystal structure in the $P4_3$ space group (PDB 7KQO, white sticks) with the open state crystal structure (teal sticks). The Phe132 side chain is shown with transparent yellow spheres. The Phe132 loop adopts a distinct conformation in the most potent open state binder (AVI-3731, IC_{50} = 3.7 μM), where the Phe132 side-chain makes contact with the indane portion of the ligand and the Phe132 loop forms a short α-helix. (**B**) Same as (**A**) but showing open-state structures reported in (*2*). Although Z8539_0025 stabilizes a similar short α-helix compared to AVI-3731, the Phe132 side chain is solvent exposed instead of contacting the ligand.

Table S1.

Summary of compound selection and filtering for FrankenROCS pipelines.

Pipeline	Step	Action	Criteria	Number of Ligands
FrankenROCS (Enamine HTS collection)	1	Initial x-ray structures		235 complexes
	2	Align to 5RS7		235 complexes
	3	Assign bond orders and extract ligands		242 ligands
	4	Calculate distances between ligand centers	< 20 Å + no overlap	7181 pairs
	5	FastROCS w/Enamine HTS	TC ≥ 1.3	692 ligands
	6	Manual selection		39 ligands
FrankenROCS with Thompson sampling (TS-FrankenROCS (Enamine REAL database)	1	Enamine REAL	97 queries with Thomson sampling	22,000,000,000
	2	TS ROCS	TanimotoCombo ≥ 1.35	25,214
	3	Energy minimize complexes		25,214
	4	Filter torsions	Total ≤ 6 and Single ≤ 1.8	6,719
	5	Filter RMS and strain	RMS ≤1.5 and Energy < 0	1,791
	6	Interaction fingerprint	Tversky similarity > 0.9	447
	7	Substrate overlap	Overlap < 0.85	221
	8	Functional group filters	Filtering rules	181
	9	Clustering	Taylor Butina cutoff 0.65	36

Table S2.

Summary of compounds tested in this work. Excel spreadsheet.

Table S3. Solubility, permeability and mouse microsomal metabolic stability for AVI-313 analogs described herein, and for previously described lead compounds AVI-92 and AVI-219.

Compound	**AVI-92***	**AVI-219***	**AVI-3669**	**AVI-1504**	**AVI-3761**	**AVI-607**	**AVI-3716**
Structure	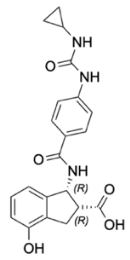	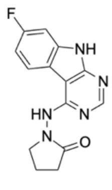	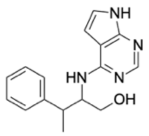	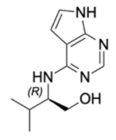	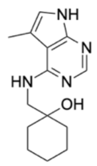	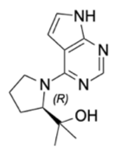	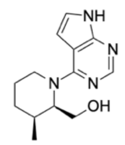
PDB code	5SQJ	5SRY	7HEC	9D6H	7HF1	9D6B	9D6G
HTRF IC_{50} (µM)	0.5	1.2	8.7	9.8	13	5.1	4.7
K_i (µM)	0.5	1.1	8.1	9.1	12	4.7	4.4
M.W. (g/mol)	395.4	285.3	282.4	220.3	260.3	246.3	246.3
Heavy atoms	29	21	21	16	19	18	18
Ligand efficiency†	0.30	0.39	0.33	0.44	0.36	0.41	0.41
Caco-2 P_{app} A-B (10^{-6}cm/s)	0.04	10.6	19.4	14.9	21.5	22.0	25.9
Caco-2 P_{app} B-A (10^{-6}cm/s)	1.7	16.8	14.5	11.3	11.2	15.7	18.9
Caco-2 ratio B-A/A-B	40.7	1.6	0.7	0.8	0.52	0.7	0.7
Mean solubility (µM)	524	127	529	370	555	518	523
Mouse microsome $T_{1/2}$ (min)	>120	26.1	8.3	>120	7.49	19	31.2
CLint (mouse) (µl/min/mg protein)	<11.6	53.1	166.4	<11.6	185	73	44.4

*Reported in (*2*)

†Binding energy per heavy atom.
